# Increased fucosyl glycoconjugate by *Mycoplasma hyopneumoniae* enhances adherences of *Pasteurella multocida* type A in the ciliated epithelial cells of the respiratory tract

**DOI:** 10.1186/s12917-016-0650-7

**Published:** 2016-02-03

**Authors:** Changhoon Park, Jiwoon Jeong, Ikjae Kang, Kyuhyung Choi, Su-Jin Park, Chanhee Chae

**Affiliations:** College of Veterinary Medicine, Seoul National University, 1 Gwanak-ro, Gwanak-gu Seoul, 151-742 Republic of Korea

**Keywords:** Co-infection, Enzootic pneumonia, *Mycoplasma hyopneumoniae*, *Pasteurella multocida*

## Abstract

**Background:**

The objective of this study was to elucidate the pathogenic mechanisms of how *Mycoplasma hyopneumoniae* enhances secondary *Pasteurella multocida* type A infection which leads to porcine enzootic pneumonia in infected pigs. Sixteen pigs were experimentally infected with *M. hyopneumoniae* and then euthanized at 7, 14, 21 and 28 days post inoculation. *In situ* hybridization for *M. hyopneumoniae* DNA and *Ulex europaeus agglutinin*-I (UEA-I) lectin histochemistry for fucosyl glycoconjugate, was performed in serial lung sections to determine alteration of fucosyl glycoconjugate in *M. hyopneumoniae*-infected bronchial and bronchiolar epithelium. Bacterial overlay assay was performed to determine the affinity of *P. multocida* type A with L-fucose.

**Results:**

The luminal surface of bronchial and bronchiolar epithelial cells that were stained with UEA-I always showed hybridization signals for *M. hyopneumoniae* but it was negative in the unaffected parts of the lung from *M. hyopneumoniae*-infected pigs and in lung from negative control pigs. Colocalization of *M. hyopneumoniae* and UEA-I was especially prominent in the luminal surface of bronchial and bronchiolar epithelial cells in serial section of lung. The mean number of *M. hyopneumoniae*-positive cells correlated with the mean number of UEA-I-positive cells in lungs from infected pigs throughout the experiment. All eight *P. multocida* type A isolates from naturally occurring enzootic pneumonia, bound strongly at levels of 2 μg and 5 μg of L-fucose.

**Conclusions:**

The results of the present study demonstrate that *M. hyopneumoniae* increases the L-fucose composition to enhance adherence of *P. multocida* type A to the bronchial and bronchiolar epithelial cells.

## Background

Mycoplasmal pneumonia, caused by *Mycoplasma hyopneumoniae*, is characterized by dry cough, retardation of growth, poor feeding efficiency, and susceptibility of pigs to secondary bacterial infection, especially *Pasteurella multocida* type A [1, 2]. Although *P. multocida* type A is the most common secondary pathogen in *M. hyopneumoniae*-infected pigs, experimental infection of *P. multocida* type A alone is not sufficient in inducing pneumonia and generally is asymptomatic in pigs [3, 4]. In contrast, infection by this organism followed by primary infection with *M. hyopneumoniae* exacerbates mycoplasmal pneumonia which can lead to porcine enzootic pneumonia in pigs [2, 4, 5]. The damaged ciliated epithelium and suppressed immunity by the *M. hyopneumoniae* infection are the main factors underlying the secondary *P. multocida* type A infection [1, 2]. Nonetheless, mechanisms of enhanced secondary *P. multocida* type A infection by *M. hyopneumoniae* have not been elucidated.

Bacterial adherence is an important initial step in the infection process that involves specific interaction between bacterial adhesins and host receptors [6, 7]. A variety of mucosal epithelial cell glycoconjugates and glycolipids act as receptors for respiratory bacterial pathogens [8, 9]. Therefore, altered composition of glycoconjugates as the result of mycoplasmal infection may be one factor that predisposes pigs to enhance secondary *P. multocida* type A infection in the lung. It has been reported that infection with *M. hyopneumoniae* enhances *Ulex europaeus agglutinin*-I (UEA-I), which has affinity of glycoconjugates for L-fucose residues, in ciliated epithelium of the respiratory tract in infected pigs [10]. These results suggest that *M. hyopneumoniae* infection can alter the composition of glycoconjugates to render the lungs susceptible to *P. multocida* type A infection.

In order to better understand the pathogenic mechanisms of how *M. hyopneumoniae* enhances the secondary *P. multocida* type A infection, first, the composition of fucosyl glycoconjugates in *M. hyopneumoniae*-infected bronchial and bronchiolar epithelium was examined, using *in situ* hybridization for *M. hyopneumoniae* DNA and UEA-I lectin histochemistry for fucosyl glycoconjugates. Second, the affinity of *P. multocida* type A for L-fucose was assessed using bacterial overlay assay.

## Methods

### Experimental design

A total of 32 colostrum-fed, cross-bred, conventional piglets were purchased at 14 days of age from a porcine reproductive and respiratory syndrome virus (PRRSV)- and *M. hyopneumoniae*-free commercial farm based on serological testing of the breeding herd, and long term clinical and slaughter history. All piglets were negative for porcine circovirus type 2 (Synbiotics, Lyon, France), and PRRSV, swine influenza virus and *M. hyopneumoniae* (IDEXX Laboratories Inc., Westbrook, ME) according to routine serological testing.

Pigs aged 14 days were randomly allocated into infected or control groups (*n* = 10 per group) using the random number generation function in Excel (Microsoft Corporation, Redmond, WA). At 21 days of age (0 days post inoculation, dpi), 16 pigs in the infected group were intratracheally administered a 10-ml dose of a lung homogenate of *M. hyopneumoniae* strain SNU98703 (1:100 dilution in Friis medium) at a final concentration of 10^4^-10^5^ color-changing units (CCU)/ml, as previously described [11]. No bacterial and viral pathogens were isolated from a lung homogenate of *M. hyopneumoniae* strain SNU98703. Sixteen control pigs were exposed in the same manner to uninfected Friis medium. Four pigs from each group were sedated by an intravenous injection of sodium pentobarbital and then euthanized by electrocution at 7, 14, 21, and 28 dpi as previously described [12]. Tissues were collected from each pig at necropsy. All of the methods were previously approved by the Seoul National University, Institutional Animal Care and Use, and Ethics Committee (SNU-140043-11B, date of approval 10 January 2014).

### Preparation of labeled probe

A 520-base-pair DNA fragment was used as a probe. The forward and reverse primers were 5’-GTGTATCAAAATTGCCAATC-3’ (nucleotides 851 to 870) and 5’-TCCCATAACCTTGTCTTCAG-3’ (nucleotides 1351 to 1370), respectively [13]. PCR was performed as previously described [13]. The PCR products were purified with Wizard PCR Preps (Promega Biotech, Madison, WI). The purified PCR products were labeled by random priming with digoxigenin-dUTP using a commercial kit (Boehringer Mannheim, Indianapolis, IN).

### *In situ* hybridization

Tissues were routinely fixed for 24 h in 10% neutral buffered formalin. After fixation, the tissues from each pig were dehydrated through a graded series of alcohol solutions and a xylene step and embedded in paraffin wax. Four serial sections (4 μm) were then prepared from each tissue, two being further processed for *in situ* hybridization (ISH) using a *M. hyopneumoniae* probe with and without DNase A treatment, one for lectin histochemistry using an UEA-I lectin, and one for haematoxylin and eosin (HE) staining. ISH was performed as previously described [14]. The lung tissues from pigs experimentally infected with *M. hyopneumoiniae* were used as positive controls for ISH [15].

### Lectin histochemistry

Sections were deparaffinized in xylene, hydrated through a graded series of alcohols to straight distilled water. Endogenous alkaline phosphatase was quenched with glacial acetic acid 20% for 2 min at 4 °C. Sections were treated for 1 h at room temperature with UEA-I lectin (Vector Laboratories, Burlingame, CA) at a concentration of 0.8 μg/ml in phosphate buffered saline (PBS, pH 7.2). The sections were washed three times with PBS. The sections were then immersed in labeled streptavidin-biotin (LSAB) plus alkaline phosphatase (AP) link universal (Dako Corporation, Carpinteria, CA) and incubated for 15 min at room temperature. Sections were then equilibrated with Tris-buffer (pH 9.5) for 5 min at room temperature. The final reaction was produced by immersing the sections in a solution of red substrate (Vector® Red Alkaline Phosphatase Substrate, Vector Laboratories) for 20 min at room temperature. The sections were lightly counterstained with Mayer’s haematoxylin, dehydrated through graded concentrations of ethanol and xylene, and mounted. Lectin binding specificity was tested with the following: (i) mixing the lectin with a 0.1M solution of its inhibitory sugar (L-fucose) for 20 min before performance of lectin histochemistry; and (ii) treating sections with 1 % sodium periodate prior to labeling for 10 min. Both pretreatments prevented staining. The small intestine tissues from *Escherichia coli*-infected pigs were used as positive control for lectin histochemistry of UEA-I [16]. The porcine ileal Peyer’s patches tissues were used as negative control for lectin histochemistry of UEA-I [17].

### Morphometric analysis

For the morphometric analyses of *in situ* hybridization and lectin histochemistry, 3 sections were cut from each of three blocks of tissue from lung of each pig. The slides were analyzed using the NIH Image J 1.43m program (http://image.nih.gov/ij/download.html) to obtain the quantitative data. For the analysis of *M. hyopneumoniae in situ* hybridization, 10 fields were randomly selected and slides were scored ranging from 0 (no signal detectable) to 3 (intense labeling on the surface of bronchial and bronchiolar epithelium) as previously described [18]. For the analysis of UEA-I histochemistry, 10 fields were randomly selected and slides were scored ranging from 0 (no signal detectable) to 3 (intense labeling on the surface of bronchial and bronchiolar epithelium).

### Radiolabeling of bacteria

Eight *P. multocida* type A isolates from porcine enzootic pneumonia were used for radiolabeling. Radioiodination of *P. multocida* type A was carried out as described previously [8] with slight modification. Bacteria (0.5 ml; 10^8^-10^9^ cells in 0.3 M sodium phosphate buffer, pH 6.8) were transferred to 10 x 75 mm tubes previously coated with 100 μg of Iodogen (Sigma Chemical Company, St. Louis, MO), and reacted with 1 mCi of Na^125^I (Perkin-Elmer, Boston, MA) at 4 °C for 10 min followed by a 5 min incubation at room temperature. Iodination was terminated by removing the cells followed by centrifugation, followed by three washes with 0.05 M Tris–HCl (pH 7.8) containing 0.15 M NaCl and 1% bovine serum albumin (TBS-BSA). The labeled bacteria were resuspended at a cell density of 5 x 10^7^-10^8^ cells per ml in TBS-BSA.

### Bacterial overlay assay

*P. multocida* type A binding to L-fucose (Sigma Chemical Company) was tested as previously described with slight modification [19]. L-fucose (0, 0.5, 1, 2, and 5 μg) was spotted on aluminum-backed silica gel high-performance plates (Merck, Germany). Plates were dried, dipped in hexane containing 0.1% polyisobutylmethacrylate, and air-dried. The plates were sprayed with TBS-BSA and immersed in TBS-BSA for 1 h. After excess buffer was drained from the plates, they were overlaid for 4 h with 60 μl of ^125^I-labeled bacteria (approximately 1 x 10^6^ colony forming units/ml) in TBS-BSA. Plates were washed five times with PBS to remove unbound bacteria, dried, and exposed overnight to X-ray film (Eastman Kodak, Rochester, NY) at room temperature.

### Statistical analysis

Spearman’s correlation was used to assess the relationship between *in situ* hybridization (*M. hyopneumoniae*) and lectin histochemistry (UEA-I). A value of *P* < 0.05 was considered significant.

## Results

### In situ hybridization of *M. hyopneumoniae*

**Fig. 1:**
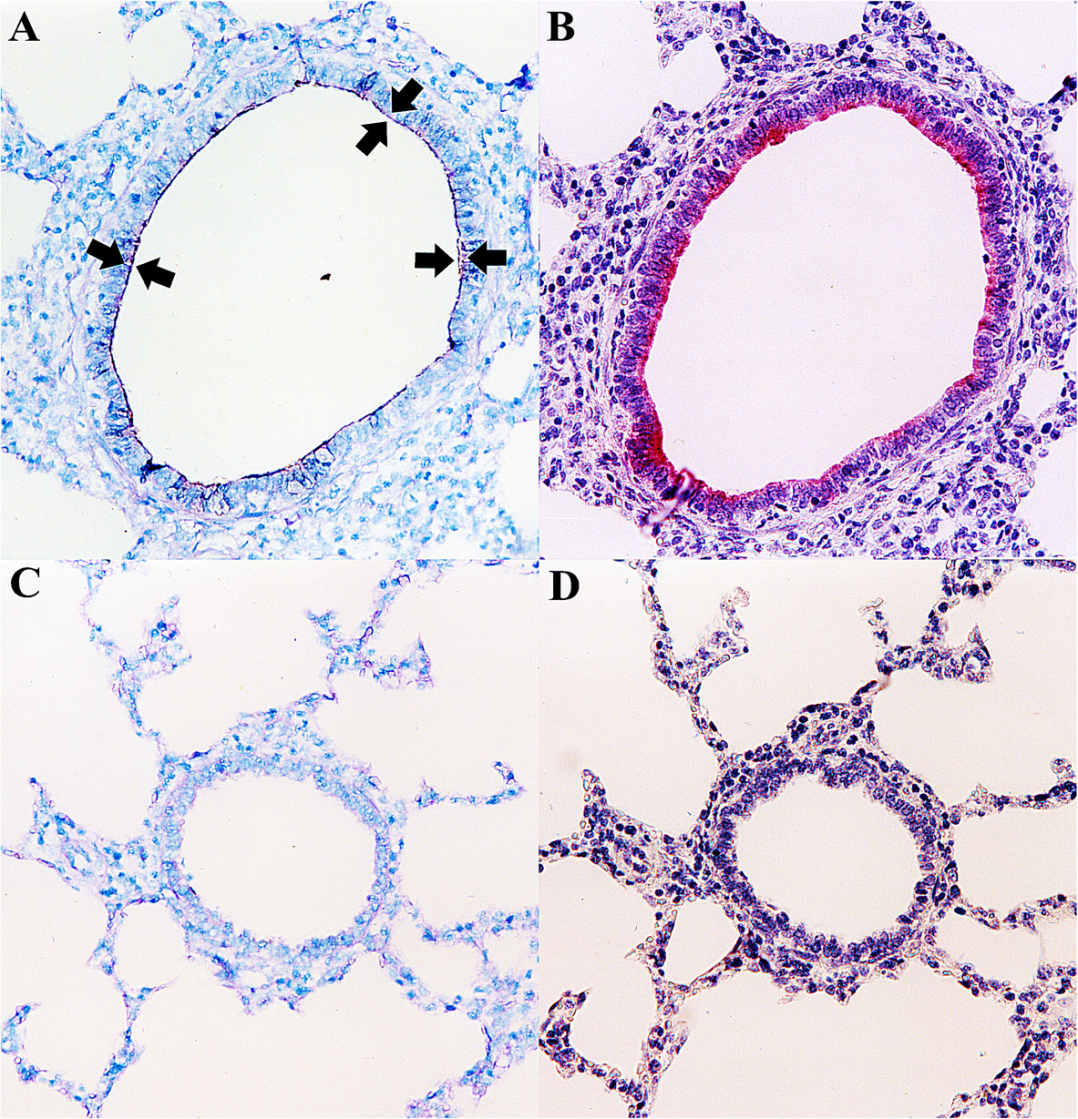
In situ hybridization and lectin histochemistry. Serial sections of the *Mycoplasma hyopneumoniae*-infected pigs at 21 dpi show that the majority of areas containing strong *M. hyopneumoniae* DNA signals (dark brown reaction, arrows; **a**) also have numerous UEA-I staining (red reaction; **b**) in the bronchial and bronchiolar epithelial cells. Serial section of the lung tissue from negative control pigs show negative *M. hyopneumoniae* DNA (**c**) and UEA-I staining (**d**) signals

**Fig. 2:**
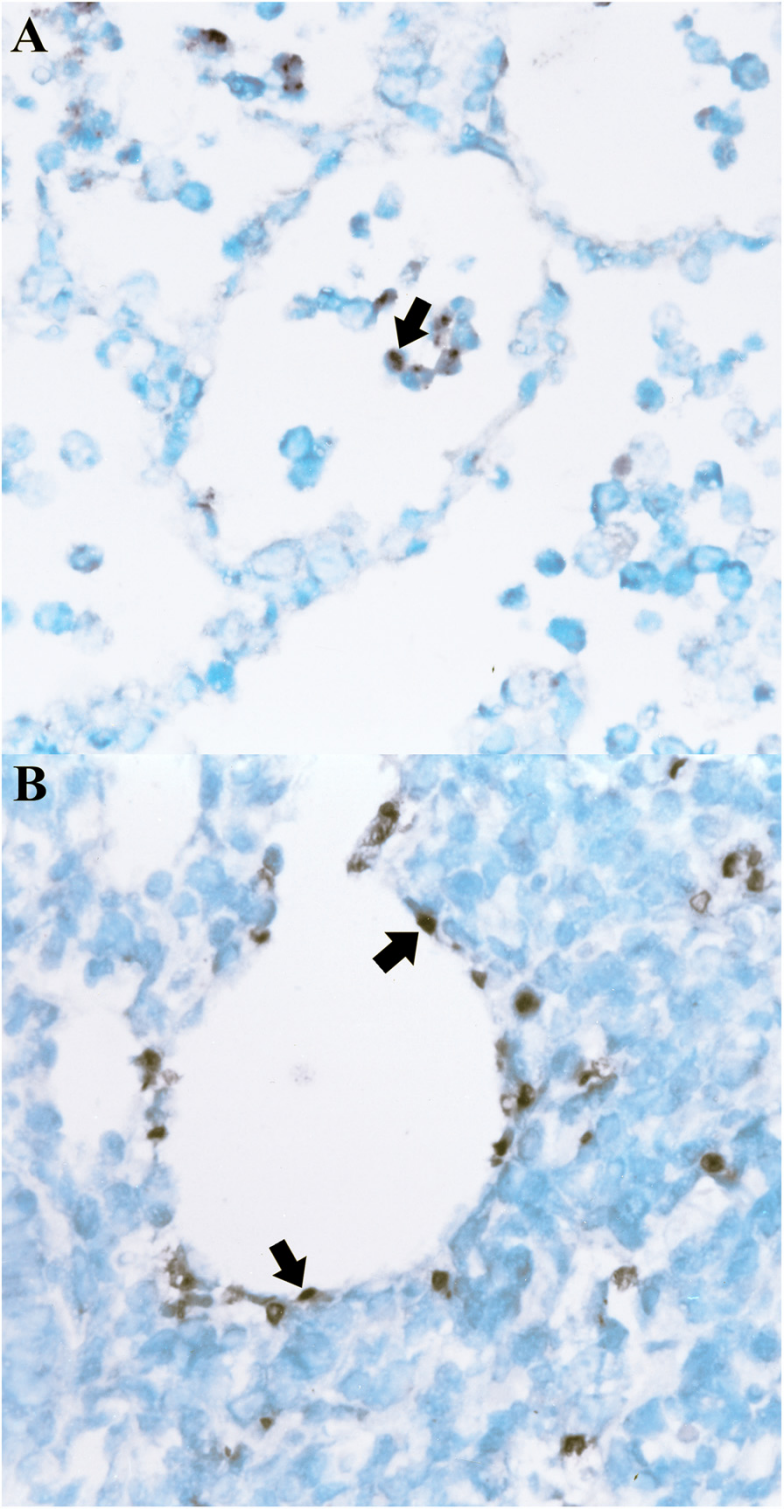
In situ hybridization. *Mycoplasma hyopneumoniae* DNA signals (dark brown reaction) are detected in alveolar macrophages (arrow, **a**) and type 1 pneumocytes (arrows, **b**) from *M. hyopneumoniae*-infected pigs at 21 dpi

The morphology of host cells was preserved despite the relatively high temperatures and chemical treatments required in the procedure. A very close cell-to-cell correlation among serial sections from each lung sample was confirmed by *in situ* hybridization. The signal intensity varied within and between histological, bronchi and bronchioles, in sections of a single animal and also between pigs. Positive cells typically exhibited a dark brown reaction protduct without background staining. *M. hyopneumoniae* DNA was detected at the luminal surface of bronchial and bronchiolar epithelial cells (Fig. 1a), alveolar (Fig. 2a) and interstitial macrophages, and type I pneumocytes (Fig. 2b) in the lung from all infected pigs at 7, 14, 21, and 28 dpi. A positive hybridization signal was especially intense at the luminal surface of bronchial and bronchiolar epithelial cells, whereas the hybridization was sparse in alveolar and interstitial macrophages, and type I pneumocytes. When a hybridization signal was detected at the luminal surface of bronchial and bronchiolar lining epithelial cells, a given bronchus or bronchiole also exhibited peribronchiolar lymphoid cuffing. Pretreatment with DNase I eliminated the hybridization signal from 16 pigs experimentally infected with *M. hyopneumoniae* and from positive control pigs. Sections from negative control pigs showed no hybridization signals for *M. hyopneumoniae* (Fig. 1c).

### Lectin histochemistry for L-fucose residues

A very close cell-to-cell correlation among serial sections from each lung sample was confirmed by lectin histochemistry. In *M. hyopneumoniae*-infected lungs, UEA-I stained the luminal surface and cytoplasm of bronchial and bronchiolar epithelial cells in the lung from all infected pigs at 7, 14, 21, and 28 dpi. Positive cells typically exhibited a red reaction product without background staining. The luminal surface and cytoplasm of bronchial and bronchiolar epithelial cells stained strongly with UEA-I in lungs from infected pigs at 14 and 21 dpi (Fig. 1b). No UEA-I staining was seen at the luminal surface or in the entire cytoplasm of bronchial and bronchiolar epithelial cells of negative control pigs (Fig. 1d). Pretreatment with L-fucose and sodium periodate eliminated histochemical staining by UEA-I in 16 pigs experimentally infected with *M. hyopneumoniae* and in positive control pigs.

### Correlation between *M. hyopneumoniae* and L-fucose residues

There was close cell-to-cell correlation when serial sections were examined by *in situ* hybridization with *M. hyopneumoniae* and lectin histochemistry with UEA-I in lung from infected pigs at 7, 14, 21, and 28 dpi. Colocalization of *M. hyopneumoniae* (Fig. 1a) and UEA-I (Fig. 1b) was especially prominent in the luminal surface of bronchial and bronchiolar epithelial cells. The luminal surface of bronchial and bronchiolar epithelial cells stained with UEA-I (Fig. 1c) always showed hybridization signals for *M. hyopneumoniae* (Fig. 1d) but it was negative in the unaffected parts of the lung from *M. hyopneumoniae*-infected pigs and in lung from negative control pigs. The score for the mean number of *M. hyopneumoniae*-positive cells per unit area of lung was correlated with the score for the mean number of UEA-I-positive cells per unit are of lung at 7 (*r =* 0.202 and *P* = 0.027), 14 (*r =* 0.522 and *P* = 0.001), 21 (*r =* 0.259 and *P* = 0.004), and 28 (*r =* 0.304 and *P* = 0.001) dpi.

### Bacterial overlay assay

**Fig. 3:**
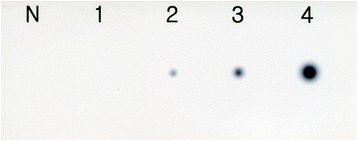
Bacterial overlay assay. Binding affinity of L-fucose detected by autoradiography with radioiodinated *Pasteurella multocida* type A. N, negative control. Lane 1, *P. multocida* type A did not bind to 0.5 μg level of L-fucose. Lanes 2–4, *P. multocida* type A bound to 1, 2, and 5 μg level of L-fucose, respectively

Eight *P. multocida* type A isolates were analyzed for their ability to bind L-fucose using bacterial overlay assay. As shown by autoradiogram, none of eight *P. multocida* type A bound to L-fucose at a level of 0.5 μg. Two *P. multocida* type A did not bind to L-fucose at a level of 1 μg whereas the remaining six *P. multocida* type A bound faintly. All eight *P. multocida* type A bound strongly at levels of 2 μg and 5 μg of L-fucose (Fig. 3). No binding was observed in negative controls.

## Discussion

The results of the present study demonstrate that increased the L-fucosyl composition in *M. hyopneumoniae*-infected pigs enhance adherence of *P. multocida* type A in the bronchial and bronchiolar epithelial cells. The numbers of *M. hyopneumoniae*-positive cells is correlated with the numbers of UEA-I-positive cells in infected cells. UEA-I staining could also be influenced by mycoplasmal enzymes; however, *M. hyopneumoniae* lack the enzyme fucosidase [20]. Therefore, UEA-I staining of the luminal surface and cytoplasm of bronchial and bronchiolar epithelial cells indicates that L-fucose may be a terminal residue on glycoconjugates synthesized by these cells in *M. hyopneumoniae*-infected pigs. In addition, eight *P. multocida* type A isolated from porcine enzootic pneumonia also showed strong affinity for L-fucose by bacterial overlay assay. A bacterial overlay assay was used to evaluate the binding of respiratory pathogens to glycoconjugates or glycolipids [8, 19]. These results suggest that increased expression of L-fucose containing glycoconjugates induced by *M. hyopneumoniae* infection may provide greater numbers of binding sites for *P. multocida* type A and subsequent infection with *P. multocida* type A results in severe enzootic pneumonia.

Increased fucosyl glycoconjugate in *M. hyopneumoniae*-infected pigs is potential factor to enhance colonization of *P. multocida* type A in bronchial and bronchiolar epithelial cells. Alternatively, *M. hyopneumoniae* colonizes the ciliated epithelial cells of the respiratory tract and damages the cells which lead to an enhanced infection of *P. multocida* type A [2]. A strong hybridization signal of *M. hyopneumoniae* was detected mainly in the luminal surface of bronchial and bronchiolar lining epithelial cells. Our results are consistent with an ultrastructural study, in which *M. hyopneumoniae* attaches to the cilia in the bronchial and bronchiolar epithelial cells [21, 22]. These results suggest that *M. hyopneumoniae* is intimately associated with the cilia and causes extensive loss of these structures. *M. hyopneumoniae* causes ciliostasis and damages to the ciliated epithelial cells of the respiratory tract [1, 21, 22], rendering the lungs susceptible to *P. multocida* type A colonization and infection.

This study explains how *M. hyopneumoniae* enhance the secondary infection of *P. multocida* type A leading to enzootic pneumonia. Altered composition of glycoconjugates as the result of mycoplasma infection may be one factor that predisposes pigs to enhance *P. multocida* type A infection in the lung. However, co-infection with *M. hyopneumoniae* and *P. multocida* type A was not conducted in this study. Therefore, further studies are needed to confirm enhanced adherence of *P. multocida* type A to ciliated epithelial cells of the respiratory tract using animal model with co-infection. Altered composition of glycoconjugates by *M. hyopneumoniae*, together with damaging to ciliated epithelial cells of the respiratory cells, generates a favorable environment promoting colonization and secondary infection of *P. multocida* type A resulting in porcine enzootic pneumonia.

## Conclusions

The objective of this study is to elucidate the pathogenic mechanisms of how *M. hyopneumoniae* enhances secondary *P. multocida* type A infection which leads to porcine enzootic pneumonia in infected pigs. Altered composition of glycoconjugates by *M. hyopneumoniae*, together with damaging to ciliated epithelial cells of the respiratory cells, generates a favorable environment promoting colonization and secondary infection of *P. multocida* type A resulting in porcine enzootic pneumonia.
